# Fabrication of Metal Contacts on Silicon Nanopillars: The Role of Surface Termination and Defectivity

**DOI:** 10.3390/ma17071549

**Published:** 2024-03-28

**Authors:** Federico Giulio, Antonio Mazzacua, Luca Calciati, Dario Narducci

**Affiliations:** 1 Department of Materials Science, University of Milano Bicocca, Via R. Cozzi 55, I-20125 Milan, Italy; f.giulio1@campus.unimib.it (F.G.); a.mazzacua@campus.unimib.it (A.M.); 2 Department of Physics ‘Giuseppe Occhialini’, University of Milano Bicocca, Piazza Della Scienza 3, I-20126 Milan, Italy; l.calciati@campus.unimib.it

**Keywords:** nanowires, nanopillars, thermoelectricity, metal-assisted chemical etching, silicon

## Abstract

The application of nanotechnology in developing novel thermoelectric materials has yielded remarkable advancements in material efficiency. In many instances, dimensional constraints have resulted in a beneficial decoupling of thermal conductivity and power factor, leading to large increases in the achievable thermoelectric figure of merit (ZT). For instance, the ZT of silicon increases by nearly two orders of magnitude when transitioning from bulk single crystals to nanowires. Metal-assisted chemical etching offers a viable, low-cost route for preparing silicon nanopillars for use in thermoelectric devices. The aim of this paper is to review strategies for obtaining high-density forests of Si nanopillars and achieving high-quality contacts on them. We will discuss how electroplating can be used for this aim. As an alternative, nanopillars can be embedded into appropriate electrical and thermal insulators, with contacts made by metal evaporation on uncapped nanopillar tips. In both cases, it will be shown how achieving control over surface termination and defectivity is of paramount importance, demonstrating how a judicious control of defectivity enhances contact quality.

## 1. Introduction

Silicon serves as a pivotal functional material; it is the bedrock for microelectronics and solar cells and is continually gaining technological significance owing to its abundance on Earth and cost-effectiveness. However, its use as a thermoelectric material is hampered by inherent limitations. Despite exhibiting relatively high power factors ($\sigma \alpha^{2}$) at elevated carrier densities, the silicon thermoelectric figure of merit $ZT=\sigma \alpha^{2}T/\kappa$ (where σ is electrical conductivity, α is the Seebeck coefficient, *T* is absolute temperature, and κ is thermal conductivity) remains meager, reaching approximately 0.01 at room temperature [1], primarily due to its high thermal conductivity of around 120 W/mK at room temperature.

To mitigate this thermal conductivity challenge without compromising electrical properties, one viable avenue involves the creation of dimensionally constrained nanostructures (NSs) [2]. Silicon nanowires (NWs) with diameters below the phonon mean-free path (MFP), ≈200 nm in Si at room temperature [3], exhibit substantially reduced thermal conductivity (<5 W $m^{-1}$ $K^{-1}$) [4,5,6]. Similarly, silicon nanolayers (NLs) also manifest lower thermal conductivity, contingent upon the nanolayer thickness [7]. In both cases, dimensional constraints cause incoherent phonon scattering at the NW/NL walls. Since, in silicon, thermal conductivity is largely due to heat transported by long MFP phonons, this leads to a major κ reduction. An alternative strategy involves top-down control to generate nanopores in ‘holey’ silicon, reducing κ without adversely affecting the power factor [8,9].

Regardless of the chosen strategy for minimizing thermal conductivity, two critical considerations must be addressed to ensure the efficacy of the resulting nanomaterial for heat harvesting or refrigeration. Firstly, manufacturing methods should facilitate the cost-effective fabrication of the final device. Equally crucial is the need to enhance efficiency (coefficient of performance) without sacrificing power density (cooling power). This is particularly pertinent in the case of NWs, where Si NWs are typically supported on insulating templates when fabricated using standard integrated-circuit technologies [10]. In most instances, however, Si NWs fabricated on insulating templates result in a low ratio of thermoelectric active cross-sections to the device footprint, significantly diminishing the accepted input thermal power and consequently limiting the output power density. We will demonstrate that metal-assisted chemical etching (MACE) can potentially surmount such limitations, concurrently addressing the imperatives of low production costs and high filling factors.

Arguably, the initial application of MACE in producing Si NWs for thermoelectric purposes was documented in one of the foundational papers on thermoelectric NWs, published back-to-back in *Nature* in 2008. In their seminal works, Boukai and colleagues achieved notable thermoelectric performance in Si NWs prepared by extreme lithography techniques [5]. Hochbaum and collaborators demonstrated comparable outcomes by employing MACE to craft NWs protruding from a silicon substrate [4]. Hereafter, we will refer to them as nanopillars (NPs). Subsequently, MACE has undergone extensive exploration, driven not only by its application in Si NPs for thermoelectricity but also due to Si NP usability in diverse areas such as anti-reflective finishing in solar cells [11], chemical sensing elements [12], and nanoantennas [13], among other examples. Compared to extreme lithography, the main advantage of MACE is its simplicity, as the method does not require high vacuum and it is easily scalable to obtain NP forests over large areas. However, NPs are randomly distributed over the Si surface, which could be an issue for specific applications.

**Figure 1:**
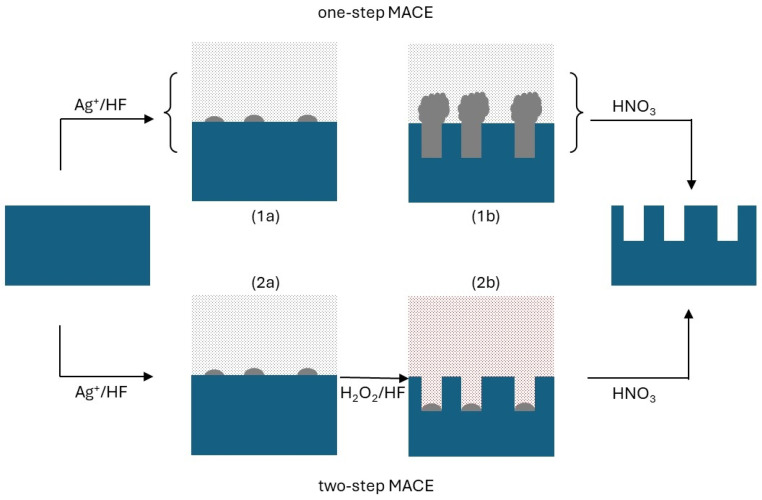
Schematics of one-pot and two-pot MACE using silver as a catalyst. In both cases, the process begins by exposing single-crystalline silicon to a solution containing ${\mathrm{Ag}}^{+}$ and HF. Silver ions are reduced to metallic silver, forming metallic nanoparticles randomly distributed on the Si surface [(1a) and (2a)]. Then, in one-pot MACE, silver keeps oxidizing silicon while the oxide is etched away by HF. Metallic silver fills the bores, also forming Ag dendrimers on top of Si (1b). In two-pot MACE, right after the nucleation of Ag nanoparticles (typically after about one minute or less), the ${\mathrm{Ag}}^{+}$/HF solution is replaced by an $H_{2}$$O_{2}$/HF solution, with hydrogen peroxide continuing Si-localized etching (2b). In both one- and two-pot MACE, the process ends with the removal of metallic Ag by ${\mathrm{HNO}}_{3}$. The unetched portion of Si forms the nanopillars. Note that, as Ag nucleation occurs randomly, the NP spacing and size are statistically distributed, although statistical dispersion is moderate.

MACE can be carried out in two ways (Figure 1). In the two-pot MACE method, silicon undergoes brief exposure to a solution containing metal ions (including ${\mathrm{Ag}}^{+}$, ${\mathrm{Cu}}^{2+}$, and ${\mathrm{Au}}^{3+}$) and HF [14]. Their reduction leads to the formation of metallic nanoparticles, sparsely coating the Si surface. The wafer is then immersed in a second solution, still containing HF and an oxidizing agent, typically $H_{2}$$O_{2}$. Silicon preferentially oxidizes at the Si–metal interface. Thus, metal nanoparticles penetrate the wafer. The remaining unetched Si materials form the NPs. Conversely, the one-pot MACE technique employs a single solution where ${\mathrm{Ag}}^{+}$ ions serve as both precursors for catalytic nanoparticles and as the oxidizing agent. In this approach as well, Si NPs are the unetched Si portions.

Although elemental metals are the most common choice as catalysts, other materials have been considered. This is especially worthwhile when NPs are to be used in microelectronics, where transition metal contamination is to be avoided. As an example, titanium nitride was shown to lead to localized etching and the formation of Si NPs, although MACE had to be performed in the vapor phase [15]. In all implementations, MACE emerged as an uncomplicated yet finely controlled method for producing single-crystalline Si NPs, affording excellent control over the wire length, orientation, doping type, and level, thereby enabling the achievement of high NP densities.

This paper reports on recent progress aimed at using MACE as a technology to obtain low-cost, large-area, efficient thermoelectric devices, exploiting the advantages of nanotechnology in the making of integrated micro-thermoelectric harvesters and macroscopic thermoelectric generators (TEGs). Specifically, it will be shown how NP forests can be obtained from both p- and n-type silicon, with doping levels up to $10^{19}$ ${\mathrm{cm}}^{-3}$, preserving their bulk crystallinity in all cases. Two procedures for establishing electrical contacts on NPs will be described. First, following an ingenious procedure developed by Pennelli and co-workers [16], it will be shown how copper can be electrochemically plated under non-equilibrium conditions, namely by applying large current densities. Contacts are always non-rectifying due to defect injections at NP tips occurring during MACE. As a second possibility, we embedded NP forests into a polymeric matrix, obtaining mechanically stabilized elements that could be used as conventional thermoelectric legs in TEGs and thermoelectric coolers (TECs). It should be mentioned that alternate approaches have been reported in the literature [17], which will be discussed and compared to electroplating and embedding.

## 2. Materials and Methods

### 2.1. One-Pot MACE

As mentioned, one-pot MACE uses ${\mathrm{Ag}}^{+}$ ions to oxidize elemental Si to Si(IV) and, once reduced to metallic Ag, catalytically localize etching underneath Ag nanoparticles decorating the Si surface. Nanoparticle diameters were measured right after nucleation to be ≈100 nm. In an exemplar implementation [18], single-crystal silicon wafers, [100]-oriented, are used. Surfaces are cleaned and the native oxide is etched using a 5 mol. % HF solution. Chips are then dried and immediately soaked into the MACE solution, which is obtained by mixing ${\mathrm{AgNO}}_{3}$ in HF to obtain final concentrations of 16 mM and 5 M, respectively. The solution is then kept at a fixed temperature (typically between 5 and 30 °C). At the end of the process (lasting up to several hours), silver dendrites filling Si bores are removed by oxidizing them with ${\mathrm{HNO}}_{3}$, followed by an accurate rinsing in deionized water. The whole process is carried out in ambient air.

### 2.2. Two-Pot MACE

Two-pot MACE makes use of different chemicals to localize the etching process on the Si surface and to oxidize Si. Several metals can be used as catalysts, including Ag, Au, and Cu [14]. In all cases, after preparing the Si surface as just reported, the wafer is exposed to a solution containing the metal ion, which reduces to its elemental state by oxidizing Si. Also in this case Ag nanoparticle diameters are ≈100 nm. The process lasts from a few seconds to a few minutes, which is enough time to obtain the needed density of metal nanoparticles. The chip is then removed from the solution and soaked into the MACE solution, which contains HF and an oxidizing agent. Typically, $H_{2}$$O_{2}$ is used at concentrations between 0.1 and 0.8 M [19]. Si oxidation preferentially occurs at the metal–Si interfaces. As a result, metal nanoparticles sink into the Si wafer. At the end of the process, metal nanoparticles are chemically removed by using a suitable oxidizing agent (${\mathrm{HNO}}_{3}$ for Ag and Cu, *aqua regia* for Au).

### 2.3. Post-MACE Processing

Both in one- and two-pot MACE, the last step after MACE is the removal of the residual metal nanoparticles/dendrites by an oxidizing solution. This final step is critical since it implies that the final drying of the Si chip commonly occurs from an aqueous solution. Since the oxidizing agent causes the formation of a thin oxide layer on the Si NP surface, the final result is that the (flexible) NPs bundle up due to capillary forces [20,21], forming tip aggregates. Bundling may be avoided by replacing spontaneous drying with super-critical drying [22,23]. As a simpler alternative, either water is replaced with less polar solvents or the NP surface oxide is etched by HF as a last step before drying [21], avoiding any capillary-driven tip agglomeration of nonpolar (hydrogenated) NP surfaces. In some cases, however, NP bundling may be desired and strengthened by willingly oxidizing NP surfaces (e.g., with $H_{2}$$O_{2}$).

### 2.4. Nanopillar Plating

After the removal of the native oxide layer, two thin layers of Cr (15 nm) and Cu (25 nm) were thermally evaporated. Chromium acts as a barrier to prevent Cu diffusion into Si while enhancing the adhesion of Cu. The thin Cu layer instead promotes the electrochemical growth of the bulk copper contact, which was deposited using three-electrode cells.

**Figure 2:**
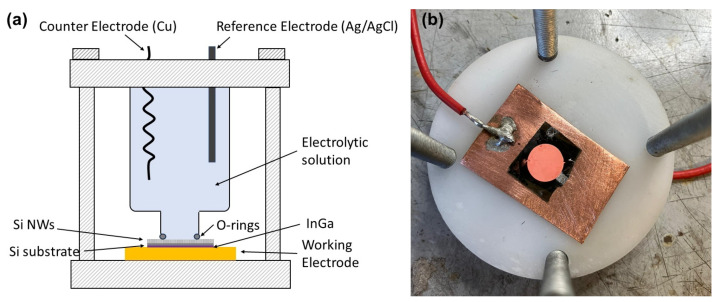
Electroplating of NP forests: (**a**) schematics of the electrochemical cell; (**b**) exemplar chip after contact fabrication.

The sample was anchored at the bottom of the cell with an O-ring seal and was connected to the cathode of the potentiostat through a copper plate (working electrode). As the counter-electrode (anode), a 1 mm thick copper coil was used, while the reference electrode was a standard Ag/AgCl electrode. The cell was filled with an electrolytic solution of ${\mathrm{CuSO}}_{4}$ (0.4 M) and $H_{2}$${\mathrm{SO}}_{4}$ (1 M). The current (25 mA for 25 min, with a current density of 312 A/$m^{2}$) was applied between the working and counter electrodes while the cell voltage was read between the working and reference electrodes. The total copper mass deposited was 12.35 mg with a total charge of 39 C. Metal deposition occurred under the galvanostatic control, setting the current density to a high value so that the applied voltage largely exceeded the equilibrium redox potential. This caused Cu reduction to take place only on NP tips. The schematics of the cell along with a picture of an NP forest after contact fabrication are displayed in Figure 2. Further details on the process have been reported in previous publications [16,24].

### 2.5. Encapsulation

**Figure 3:**
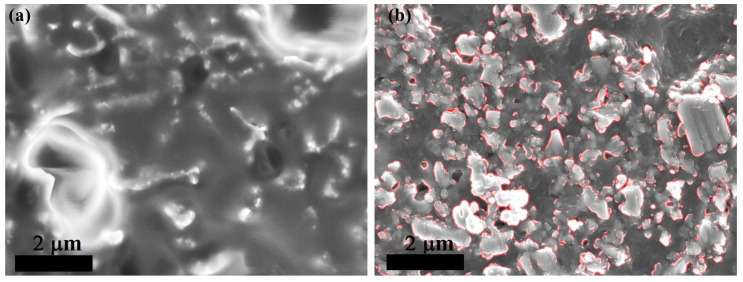
Top-view micrographs of polymer-embedded NPs: While (**a**) NP tips are fully coated by the polymer upon embedding, mechanical polishing (**b**) causes a large fraction of NP tips (highlighted by red contours) to be uncapped.

Encapsulation was initiated by depositing 50 μL of hydroxypropyl methacrylate (HPMA) on 1.5×1.5 ${\mathrm{cm}}^{2}$ samples. During the impregnation process, samples were kept under vacuum (residual pressure of 20–50 mbar) to prevent the inactivation of the initiator, $2,2^{\prime }$-Azobis(2-methylpropionitrile), by oxygen. Then samples were heated for ten minutes at 90 °C, still under vacuum, using a hot plate. At the end of the polymerization process, samples were fully encapsulated by the polymer and the sample surface was electrically insulating. To expose NP tips, the excess polymer was removed by abrasive polishing to a final roughness of ≤0.3 μm. By the end, the top surface was probed using test leads, reporting low-resistance paths across exposed NP tips. Figure 3 comparatively displays the surface before and after polishing. Tips are visible in both micrographs, being fully covered by the polymer upon encapsulation, which is instead mostly exposed after polishing.

Metal contacts were finally deposited (15 nm of Cr and 500 nm of Cu) by e-beam evaporation.

### 2.6. Electrical Characterization

The same setup was used for both electroplated and encapsulated samples. Sample current–voltage characteristics were measured in a two-probe configuration using a Series 2400 source meter (Keithley Instruments, Cleveland, OH, USA). Samples were placed on a copper plate acting as the live electrode. The back sides of the samples were covered with an eutectic In–Ga alloy to reduce contact resistance. The top copper contact acted instead as the ground electrode.

## 3. Results

Unless otherwise stated, the results reported in this Section refer to NPs obtained by one-pot MACE.

### 3.1. Localized Electroplating

Devices obtained by electroplating NP forests display non-rectifying metal-tip contacts for any doping level and type. Rectification occurred at the back contact at low doping. Extended defects due to strain occurring during Si extrusion [18] along with vacancy injected upon Si oxidation caused a large density of surface states at NP tips, pinning the Fermi level and leading to non-rectifying tunnel junctions. The surface state density is much lower at Si back contacts, where Schottky barriers form. To confirm this ansatz, back contacts were mechanically damaged (scratched), injecting surface states. Linear current–voltage characteristics were recorded at any doping level.

**Figure 4:**
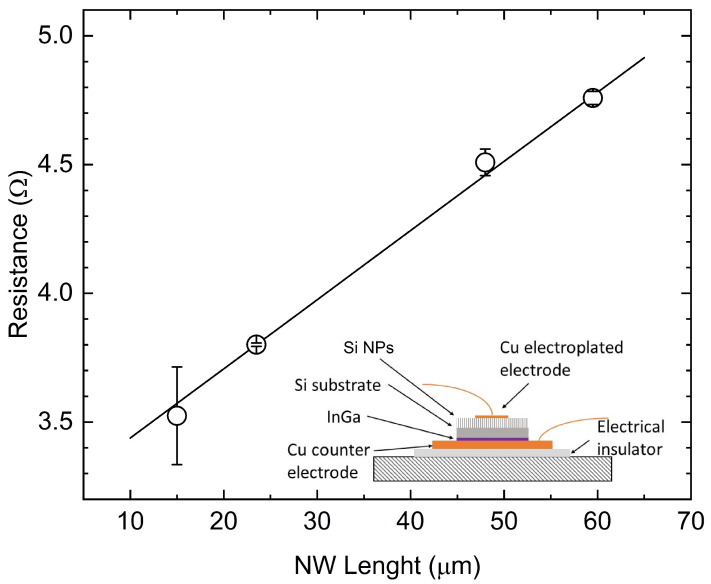
Typical dependence of the electrical resistance upon the NP length in an electroplated device. Data refer to the p-type Si and the doping level of $10^{15}$ ${\mathrm{cm}}^{-3}$. The inset shows a schematic of the measurement cell.

For the localized electroplating to be of use during the making of NP-based thermoelectric devices, short circuits between contacts must be avoided. In principle, electroplating might cause such shorts to occur, with metal deposited onto NP walls. To verify the absence of short circuits, device resistances, *R*, with changing NP lengths, *ℓ*, were measured (Figure 4). They report a linear dependence. Furthermore, apparent resistivity, defined as $\rho_{\mathrm{app}}=R\left(A/l\right)$ (where *A* is the footprint area), is found to be higher than that of the pristine material. Thus, one may conclude that there is an absence of continuous metal paths between Cu top contacts (grown onto NP tips) and the dips among NPs. Contact resistance $R_{c}$ was found to be <1 $\Omega /{\mathrm{cm}}^{2}$.

On these bases, and presuming that NPs retain the resistivity of the pristine bulk silicon ρ [4], resistance may be used to estimate the NP filling factor after plating. The resistance is expressed as follows:

(1) $$R=R_{c}+\rho \frac{s-l}{A}+\rho \frac{l}{Af_{e}}$$

where *s* is the wafer thickness, the *electrical* filling factor $f_{e}$ is found to be 0.307±0.02 for p-type Si, with a boron density of $10^{15}$ ${\mathrm{cm}}^{-3}$. *Visual* filling factors, counting the areal fraction of in-plane SEM micrographs covered by tip ends, computes instead to 0.39±0.03. For comparison, prior to contact deposition, visual filling factors account for 0.35±0.02 for bundled NPs and 0.43±0.04 for unbundled NPs.

### 3.2. Contact Metallurgy on Encapsulated Nanopillars

Encapsulation of the NP forest is effective when it mechanically strengthens the structure while not misaligning NPs. Additionally, the removal of the capping layer must enable contacts to be established on the NP tips.

**Figure 5:**
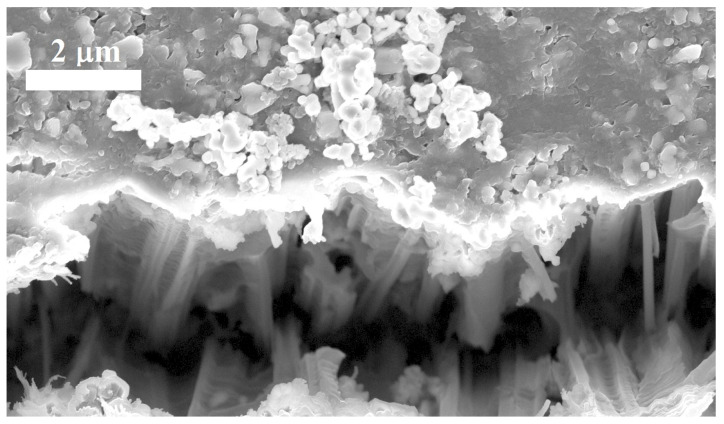
Encapsulated Si nanopillars: Fractured polymer shows how NPs retain their alignment after polymerization.

**Figure 6:**
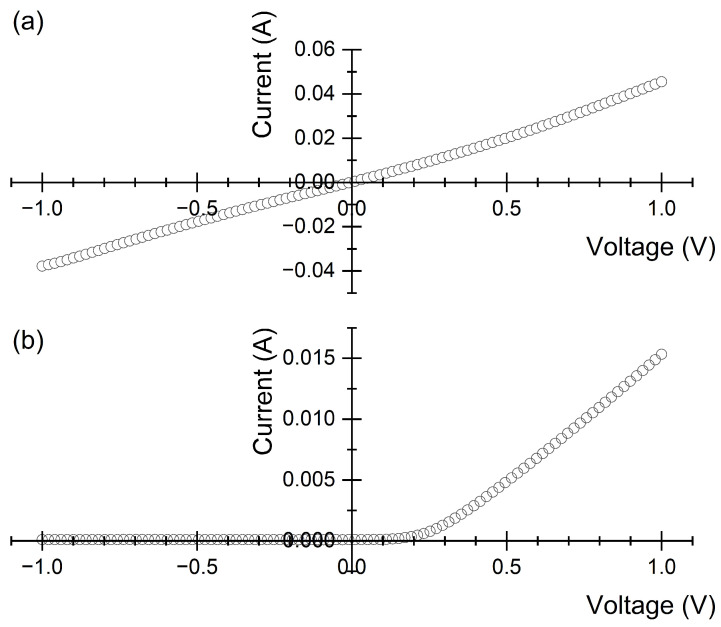
Current–voltage characteristics of (**a**) n–type and (**b**) p-type encapsulated Si NPs, $10^{15}$ ${\mathrm{cm}}^{-3}$ doping. The top contact is grounded.

Figure 5 displays a cross-section of encapsulated NPs, confirming that the exposure of NPs to HPMA does not disrupt their pristine preferential alignment. Current–voltage characteristics were measured on both n- and p-type Si NPs at the lowest doping level ($10^{15}$ ${\mathrm{cm}}^{-3}$). In the p-type samples, a Cu thin film was deposited by e-beam evaporation to establish the front contact (on NP tips) while the back contact was obtained by using In–Ga. We observed the formation of Schottky barriers at contacts. This is apparently consistent with the explanation provided for the current–voltage characteristics of electroplated systems. In the present case, while mechanical damage could avoid rectification at the back side, mechanical polishing removed the pristine, defective tip surface. Thus, no pinning of the Fermi level occurred, causing a barrier to form at the metal–tip junction that led to a rectifying contact (Figure 6). In n-type samples, where front contacts were made using Al, current–voltage characteristics were linear as expected for n–Si/Al junctions. A visual filling factor of 0.26±0.02 was measured. It compares well to an electrical filling factor of 0.28±0.04 with a contact resistance of 180 Ω/${\mathrm{cm}}^{2}$.

## 4. Discussion

In this section, we first summarize the mechanism of MACE, emphasizing the model that explains bore protection during one-pot MACE. Such a mechanism is a key element used to explain the observed characteristics of metal contacts.

### 4.1. Electrochemistry of MACE

In either one- or two-pot implementation, MACE involves an electrochemical reaction (reduction of the oxidant and the oxidation of Si) and a chemical process, namely the etching of oxidized silicon. The electrochemistry of MACE has been addressed by many scholars. The use of $H_{2}$$O_{2}$/HF solutions on surfaces that were previously patterned either with Ag or Au has received the most substantial attention [25,26,27,28], as it may also be used to fabricate ordered nanopillar arrays. In many contexts, however, randomly distributed NPs are of use, and one-pot MACE provides an easier way to obtain NP forests. In both cases, the anodic reactions of the electroless process are the same [29]. The oxidant injects holes into Si, where two processes take place. In the first, silicon is directly oxidized to ${\mathrm{SiO}}_{2}$ as

(2) $${\mathrm{Si}}_{\left(s\right)}+4h^{+}+6H_{2}O_{\left(\mathrm{aq}\right)}⟶{\mathrm{SiO}}_{2\;(s)}+4H_{3}O_{\left(\mathrm{aq}\right)}^{+}\;E{}^{\circ}=-0.84V\;vs.\;NHE$$

and then ${\mathrm{SiO}}_{2}$ is etched by HF:

(3) $${\mathrm{SiO}}_{2(s)}+6{\mathrm{HF}}_{\left(\mathrm{aq}\right)}⟶{\mathrm{SiF}}_{6\;(\mathrm{aq})}^{2-}+2H_{3}O_{\left(\mathrm{aq}\right)}^{+}$$

Thisprocess is often referred to as electropolishing. When etching occurs homogeneously over the whole surface area, it results in a smooth, crystalline, defect-free surface. When etching is localized, it leads to the formation of single-crystalline NPs.

A two-electron process may also occur, where Si is initially oxidized to Si(II) and dissolved as such [30]:

(4) $${\mathrm{Si}}_{\left(s\right)}+2h^{+}+4{\mathrm{HF}}_{\left(\mathrm{aq}\right)}+4H_{2}O_{\left(\mathrm{aq}\right)}⟶{\mathrm{SiF}}_{4\;(\mathrm{aq})}^{2-}+4H_{3}O_{\left(\mathrm{aq}\right)}^{+}\;E{}^{\circ}=-1.2VV\;vs.\;NHE$$

Oxidationof Si(II) is then completed in the solution:

(5) $${\mathrm{SiF}}_{4\;(\mathrm{aq})}^{2-}+2{\mathrm{HF}}_{\left(\mathrm{aq}\right)}⟶{\mathrm{SiF}}_{6\;(\mathrm{aq})}^{2-}+H_{2\;(g)}$$

also with the formation of $H_{2}$. This second mechanism always results in the formation of a porous silicon layer or, when localized, silicon NPs with porous surfaces [30].

As of the cathodic reaction, in one-pot MACE, ${\mathrm{Ag}}^{+}$ ions are reduced to Ag

(6) $${\mathrm{Ag}}_{\left(\mathrm{aq}\right)}^{+}⟶{\mathrm{Ag}}_{\left(s\right)}+h^{+}\;E{}^{\circ}=+0.79V\;vs.\;NHE$$

either at the bare Si surface or at the Ag surface. They are the only oxidants sustaining the redox reaction. In two-pot MACE silver, nanoparticles are nucleated in the first stage, and then Si is typically oxidized by hydrogen peroxide, as follows:

(7) $$H_{2}O_{2(\mathrm{aq})}+2H_{\left(\mathrm{aq}\right)}^{+}⟶2H_{2}O_{\left(\mathrm{aq}\right)}+2h^{+}\;E{}^{\circ}=+1.77V\;vs.\;NHE$$

The literature indicates that the two processes take place simultaneously during the electrochemical etching of silicon. In an electrochemical cell, electropolishing becomes dominant at elevated current densities, surpassing a critical current density, $j_{\mathrm{PS}}$, of approximately 0.5 A/${\mathrm{cm}}^{2}$ [29].

The role of the threshold current can be explained by considering the rate-limiting step of the electrochemical etching of silicon. At lower potentials, the rate of oxide formation is slow, leading to the immediate etching of ${\mathrm{SiO}}_{x}$ as it forms. Consequently, local oxide passivation of the surface (resulting in porous Si through the two-electron mechanism) occurs when oxide formation proceeds slowly, namely the oxidation rate is minimal. Rather, at a higher potential, oxide accumulates across the entire surface, as its removal by HF is now a slow step. Thus, the entire surface is uniformly coated with the oxide, and its removal by HF occurs simultaneously across the entire surface. This results in Si electropolishing.

A comprehensive examination of the intricate interplay between the defectivity of NP surfaces and doping levels and types was carried out [18]. Transmission electron microscopy investigation, aligning partially with prior studies [26,31,32], reveals that at low doping levels (either a p-type or n-type), NPs maintain their crystallinity without any discernible defect. Conversely, at higher doping levels (both n- and p-types), surface damage becomes evident. It is crucial to emphasize that in the context of one-pot MACE, only the outer surfaces of NPs exhibit porosity, with the bulk of the NPs retaining their pristine crystalline structures. This observation lines up seamlessly with the underlying etching mechanism. As the NPs constitute the unetched segment of the original wafer, the increasing influence of the two-electron mechanism exclusively impacts the portion of NPs exposed to the etching solution, resulting in porosity solely on the outer surface while the bulk retains its crystallinity.

It is intriguing to note that, as dopant concentrations increase, irrespective of whether they are of p- or n-types, the etch rates exhibit a decay. The activation energy of MACE undergoes a decrease when transitioning from $p^{++}$ to $n^{++}$ substrates [18]. Additionally, surface porosity manifests even in lightly doped substrates at elevated MACE temperatures, where etch rates (or injected current densities) are comparatively higher. The parallel trend observed in etch rates and the prevalence of two-electron over four-electron processes suggest that there is no simple relationship between etch rates and surface porosity. For a deeper understanding of the hole injection mechanism, delving into the electronic structure of Si and Ag, along with their interface, proves insightful. Considering that polycrystalline Ag deposited onto silicon at low temperatures yields an experimental Ag work function as low as 4.26 eV [33], the existence of a barrier opposing hole injection becomes questionable. It appears that the crucial determinant for hole injection through a non-rectifying contact is the material resistivity, ρ, governing the electromotive force, E experienced by Si. Since E=jρl (with *ℓ* denoting the distance between cathodic and anodic regions) and acknowledging that the current density, *j*, is proportional to the etch rate, it is evident that surface-porous NPs are achieved only under low voltage conditions. This observation agrees with the findings of Zhang and co-workers [34] concerning the electrochemical formation of porous silicon. The impact of MACE temperature also aligns with this model, as higher temperatures escalate the rate of the chemical step in the etching process. Consequently, the range of potential differences where HF removal is not the limiting reaction step widens, leading to the observation of surface-porous NPs at higher E.

### 4.2. Etching Localization and Lateral Bore Protection

A pivotal aspect defining any MACE process is the localized etching of Si. On (100) Si surfaces, etching rates remain uniform across all {100} surfaces. Consequently, one might anticipate lateral bore etching as holes injected into Si diffuse to neighboring regions. However, this expectation is contradicted, as protective mechanisms—either chemical or electrochemical—are in effect.

**Figure 7:**
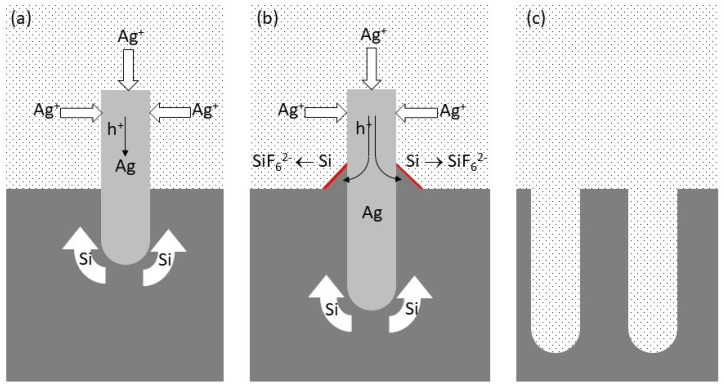
Schematics of the MACE mechanism: (**a**) Silver reduction simultaneously injects holes and transfers momentum to the growing Ag dendrimer; (**b**) dendrimer pressure causes the penetration of Ag into Si and Si extrusion (flakes), where silicon oxidizes and ${\mathrm{SiO}}_{2}$ is then removed by HF; (**c**) by the end, after Ag removal, NPs remain. Note that the NP tips are the regions where most of the mechanical stress, induced by Ag-triggered extrusion, accumulates throughout the duration of the MACE process.

In the case of two-pot MACE, bore protection arises from the heightened etching rate at the metal–silicon interface. As metal nanoparticles embed themselves into Si, etching primarily occurs at the bottom of the bores [31]. Conversely, in one-pot MACE, bores are overfilled with metallic silver. Pal, Ghosh, and Giri [35] attributed bore protection to the decreased stability (higher reactivity) of Si surfaces at the bottom of the bore, induced by defects stemming from the ongoing oxidation process. Wall protection by hydrogen released through two-electron MACE has also been considered, although this mechanism is evidently unsuitable for explaining directional etching leading to single-crystalline NPs [36]. An alternative mechanism, based on the analysis of Ag particle dynamics in $H_{2}$$O_{2}$/HF MACE, offers a fresh perspective. Drawing inspiration from a model proposed by Peng et al. for two-pot MACE [37], we posited [18] that diffusion hindrance by Ag dendrimers prevents ${\mathrm{Ag}}^{+}$ hole transfer at the submerged end of the Ag aggregate. Consequently, the silver reduction must take place at the outer dendrimer end, propelling momentum transfer at the solution-Ag interface. This process causes Si to be extruded at the surface, where oxidation takes place (Figure 7). This dual functionality explains both the directional etching (and, hence, the lateral bore protection in one-pot MACE) and the formation of Si flakes enveloping Ag nanoparticles [18].

### 4.3. Filling factors and Contact Resistances

It should be stressed that any attempts to visually count tips and their areas are subjected to large incertitude; however, the agreement between electrical and visual filling factors lend credibility to each other.

Comparable filling factors before and after contact formation show that both approaches—electroplating and polymer encapsulation—caused no significant alteration of NP orientation. This is not obvious, as both methodologies exposed NP forests to chemicals that could have modified their arrangement. Concerning contact resistances, at present, electroplating outperforms polymer embedding. Residual polymer layers on top of NP ends that survive polishing can be ruled out since insulating layers between Si and the metal layer would lead to non-linear current–voltage characteristics independently of the doping type. It cannot be excluded that, during polymerization, the polymer might also partially cover the sample’s backside, decreasing the real back-contact area and, therefore, introducing a large apparent contact resistance. Nonetheless, embedding NPs remains a key issue in granting due mechanical stability to thermoelectric legs. Therefore, additional research will be needed to optimize the process.

### 4.4. Nanopillar Defectivity

Comparison between current–voltage characteristics of electroplated and encapsulated devices based on lightly doped NPs ($10^{15}$ ${\mathrm{cm}}^{-3}$), and p- and n-types, provide insights about their defectivity.

First, the striking difference between contacts made with Cu on encapsulated or electroplated p-type NPs is consistent with the mechanism of NP formation by MACE. Although MACE implies faster etching at the metal-Si interface, etching also occurs over the clean Si surface. Both two- and four-electron etching mechanisms inject defects into Si-inducing localized states at the Si-${\mathrm{SiO}}_{x}$ interface (as detected by photoluminescence spectroscopy [38]). Their formations sensibly result from the strain generated during MACE. As reported, in one-pot MACE, the anodic process takes place at the extruded Si flakes, where strain accumulates and exceeds the elastic regime, causing the formation of point and extended defects. Localized states associated with such surface defects explain the lack of rectification in electroplated samples. Tunneling through a thin potential barrier at the metal–semiconductor junction is responsible for the linear current–voltage characteristics. This is also in agreement with the initial rectifying back-contact, which could be made linearly by injecting defect states through mechanical damage.

When encapsulated, polishing needed to uncap NP ends results in the removal of the pristine tips. Thus, the top surface exposed to the metal by NPs was never subjected to any chemical etching and is, therefore, virtually defect-free, since polishing (down to a residual roughness of 0.3 μm) on bulk silicon has been shown not to introduce a significant density of localized states on the surface.

The picture for n-type NPs is completely different. Aluminum contacts (with an Al work function of 4.08 eV) are sufficiently electropositive to avoid setting any barrier on n-type Si. Thus, both electroplated and encapsulated NPs display ohmic characteristics.

## 5. Conclusions

We presented and discussed strategies to obtain metal contacts on Si nanopillars prepared by metal-assisted chemical etching. Two methodologies were considered, namely direct electroplating, where copper contacts were fabricated using electrochemical deposition, and polymer encapsulation followed by the standard e-beam-assisted evaporation of metal contacts. In both cases, we showed how optimization of the preparation conditions is strictly entangled with accurate control of the NP defectivity and factors ruling their mutual interactions. To this aim, results achieved over the years by the present authors and by other scholars were summarized. Building upon such findings, we showed how NP surface termination rules NP bundling, not only at the end of the MACE process but also in the subsequent stages of contact fabrication. This enabled the preparation of contacted NP forests preserving high filling factors, which is a prerequisite for using NP forests as active components (legs) in TEGs and TECs.

When contacts were obtained by electroplating, low contact resistances could be obtained along with non-rectifying metal-NP junctions on both n- and p-type NPs at any doping level. Additional work is needed when NPs are embedded into an insulating matrix. While the filling factor remains large, contact resistance is large, possibly because of the improvable polymerization and polishing procedures. As an alternate strategy, Amat et al. reported on the successful fabrication of metallic contacts on polymer-embedded NPs by using reactive-ion etching (RIE) to uncap NP tips [17]. Upon accurate optimization of RIE conditions (power, duration, and cycles), $O_{2}$ plasma enabled the selective removal of the polymer (a photolithographic resist) while preserving Si NPs. Although RIE is a technology that is suitable for processing large-area samples, the last step of contact deposition was reported to require the use of (non-scalable) e-beam lithography. Nonetheless, replacing or complementing mechanical polishing with oxidative etch-back is an intriguing approach that should be further explored.

Concerning contact linearity, mechanical polishing that uncaps NP ends also removed the pristine NP tips, where defects were injected upon MACE processing. Thus, paradoxically enough, the lower defectivity of the metal–silicon interface did not allow for obtaining non-rectifying contacts on p-type NP forests. Strategies, including low-temperature metal alloying, to obtain tunneling linear junctions on p-type NPs are under evaluation.

In all cases, we showed how the wise control of defectivity is of paramount importance to understanding and controlling contact quality toward the full qualification of NP forests as Si-based thermoelectric legs with high figures of merit to be deployed in thermoelectric devices.

## Data Availability

Data are contained within the article.

## Abbreviations

The following abbreviations are used in this manuscript:

MACE	metal-assisted chemical etching
MFP	mean-free path
NP	nanopillar
NS	nanostructure
NW	nanowire
RIE	reactive ion etching
TE	thermoelectric
TEC	thermoelectric cooler
TEG	thermoelectric generator
